# The ACE2/Apelin Signaling, MicroRNAs, and Hypertension

**DOI:** 10.1155/2015/896861

**Published:** 2015-03-01

**Authors:** Lai-Jiang Chen, Ran Xu, Hui-Min Yu, Qing Chang, Jiu-Chang Zhong

**Affiliations:** ^1^State Key Laboratory of Medical Genomics, Ruijin Hospital, School of Medicine, Shanghai Jiao Tong University, Shanghai 200025, China; ^2^Shanghai Key Laboratory of Hypertension, Shanghai Institute of Hypertension, Shanghai 200025, China; ^3^Institute of Health Sciences, Shanghai Institute for Biological Sciences, Chinese Academy of Sciences, Shanghai 200025, China; ^4^Department of Cardiology, Guangdong General Hospital, Guangdong Academy of Medical Sciences, Guangzhou 510080, China

## Abstract

The renin-angiotensin aldosterone system (RAAS) plays a pivotal role in the development of hypertension. Angiotensin converting enzyme 2 (ACE2), which primarily metabolises angiotensin (Ang) II to generate the beneficial heptapeptide Ang-(1-7), serves as a negative regulator of the RAAS. Apelin is a second catalytic substrate for ACE2 and functions as an inotropic and cardiovascular protective peptide. The physiological effects of Apelin are exerted through binding to its receptor APJ, a seven-transmembrane G protein-coupled receptor that shares significant homology with the Ang II type 1 receptor (AT1R). The deregulation of microRNAs, a class of short and small noncoding RNAs, has been shown to involve cardiovascular remodeling and pathogenesis of hypertension via the activation of the Ang II/AT1R pathway. MicroRNAs are linked with modulation of the ACE2/Apelin signaling, which exhibits beneficial effects in the cardiovascular system and hypertension. The ACE2-coupled crosstalk among the RAAS, the Apelin system, and microRNAs provides an important mechanistic insight into hypertension. This paper focuses on what is known about the ACE2/Apelin signaling and its biological roles, paying particular attention to interactions and crosstalk among the ACE2/Apelin signaling, microRNAs, and hypertension, aiming to facilitate the exploitation of new therapeutic medicine to control hypertension.

## 1. Introduction

Hypertension is a complex cardiovascular disease and contributes to worldwide morbidity and mortality, while its pathogenesis is closely related to abnormalities of the renin-angiotensin aldosterone system (RAAS) [1]. Angiotensin converting enzyme 2 (ACE2) serves as a negative regulator of RAAS and cleaves angiotensin (Ang) I to generate the inactive Ang-(1-9) peptide, which can then be converted to Ang-(1-7) by ACE [2, 3]. Meanwhile, ACE2 can directly metabolize Ang II to generate the beneficial heptapeptide Ang-(1-7), whose actions are often opposite to those attributed to the Ang II and its type 1 receptor (AT1R) signaling. Apelin, a second catalytic substrate for ACE2, has powerful positive inotropic actions and vasodilatation in an endothelium- and nitric oxide- (NO-) dependent way [4]. Intriguingly, recent studies have demonstrated that there is a link between the ACE2/Apelin signaling and microRNAs (miRNAs) in the pathogenesis of hypertension. The deregulation of miRNAs expression plays a pivotal role in the development of hypertension. The miRNAs are involved in the regulation of diverse biological processes determining cell proliferation, differentiation, migration, and apoptosis [5–7]. In this review, we focus on the relationship among the ACE2/Apelin signaling, miRNAs, and hypertension and aim to facilitate the exploitation of new therapeutic medicine to control hypertension.

## 2. miRNAs and Hypertension

The miRNAs are small double-stranded, noncoding RNAs that function as guide molecules in RNA silencing [5]. miRNAs may regulate gene expression by binding to the 3′-untranslated region (3′-UTR) of target messenger RNA transcripts, leading to suppression of protein synthesis or mRNA degradation [5–7]. Previous studies have demonstrated that the majority of miRNAs generate from introns of protein-coding transcripts or noncoding genes in canonical pathways [8]. In general, miRNAs are transcribed by RNA polymerase II as a pri-miRNA, which subsequently cleaved into mature miRNA by two RNase enzymes, Drosha and Dicer, in the nucleus and cytoplasm. miRNAs biogenesis is under stringently control and their deregulation links to various diseases [8]. The regulation of miRNAs occurs at multiple levels of their biogenesis. In the process of miRNAs transcription, miRNAs are controlled by RNA polymerase II-associated transcription factors and epigenetic regulators, such as DNA methylation and histone modifications, which are positively or negatively correlated with miRNAs expression, while in the posttranscriptional levels, the modification of miRNAs processing, including RNA editing, methylation, uridylation, adenylation, and RNA decay, contributes to miRNAs regulation. Several critical cell signaling systems have been linked with regulating miRNAs biogenesis at posttranscription levels, such as transforming growth factor-*β*/bone morphogenetic protein pathways and mitogen-activated protein kinases signaling pathways, indicating the diverse possibilities for the regulation of miRNAs biogenesis [8, 9].

miRNAs are abundant in all human cells, acting as critical regulators for major cellular functions and miRNAs deregulation associated with a variety of diseases [5, 6]. Recent studies have demonstrated that miRNAs are associated with the pathogenesis of hypertension [7]. In spontaneously hypertensive rats (SHR) model, the expressions of miR-155 and miR-208 were downregulated and negative correlated with blood pressure [10, 11]. Compared with healthy controls, the plasma levels of miR-126, miR-133, miR-143, and miR-145 were decreased in patients with essential hypertension, combined with enhanced expression of miR-1, miR-296-5p, and miR-let-7e [12–14]. Furthermore, expressions of miR-21, miR-143, and miR-145 were negatively correlated with blood pressure levels, while expressions of miR-9, miR-126, and miR-133 were positively correlated with blood pressure levels [5, 12–14].

It is well established that vascular smooth muscle cells (VSMCs) proliferation, differentiated phenotype, and migration are pivotal cellular events for the development of hypertension [15]. miRNAs are highly expressed in vasculature and modulate diverse vascular cell functions (Table 1) [5, 6]. Some specific miRNAs have been identified to be involved in VSMCs biology. MiR-143 and MiR-145 are highly expressed in VSMCs and promote VSMCs differentiation and phenotype transformation via the activation of Krüppel-like factor4 (Klf4) and Klf5 (Table 1) [7, 16, 17]. MiR-143 and miR-145 are important stimuli to maintain vascular smooth muscle contractile differentiation, while knockout of them would decrease the blood pressure levels [16]. In addition, miR-365 exerts an antiproliferative role in VSMCs proliferation via the cell cycle regulatory protein cyclin D1 (Table 1) [18]. p27(Kip1) and p57(Kip2) were 2 target genes that were involved in miR-221- and miR-222-mediated effect on VSMC growth and differentiation (Table 1) [6, 7]. These observations indicate that various miRNAs in vasculature may be linked with hypertension and provide novel pharmacologic implications for the prevention and treatment of hypertension.

## 3. The ACE/Ang II/AT1R Signaling and MicroRNAs in Hypertension

**Figure 1:**
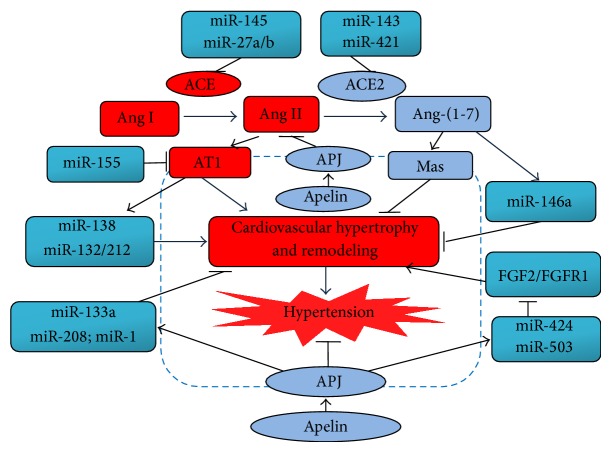
The crosstalk between ACE2/Apelin signaling and miRNAs in hypertension. On one hand, the miR-145 and miR-27a/b suppress the action of ACE/Ang II/AT1R, whereas miR-143 and miR-421 serve as negative regulators of ACE2 to modulate the balance between Ang II degradation and Ang-(1-7) generation. Overexpression of miR-155 inhibits the effects mediated by Ang II/AT1R signaling while Ang II regulates miR-138 and miR-132/212 expression via its receptor AT1R, contributing to cardiovascular hypertrophy and remodeling and elevated blood pressure. In contrast, Ang-(1-7) promotes the level of miR-146a, which blocks inflammation and cardiovascular remodeling. On the other hand, the Apelin/APJ signaling regulates the expression of miR-133a, miR-208, and miR-1, functioning as negative regulators for cardiac hypertrophy and contractile function. Moreover, the Apelin/APJ ameliorates cardiovascular hypertrophy and remodeling by modulating miR-424/-503-FGF signaling. ACE: angiotensin converting enzyme; ACE2: angiotensin converting enzyme 2; Ang II: angiotensin; AT1R: angiotensin II type 1 receptor; miRNAs: microRNAs; FGF2: fibroblast growth factor 2; FGFR1: fibroblast growth factor receptor 1.

Activation of the ACE/Ang II/AT1R signaling promotes inflammation, oxidative stress, and fibrosis linked to hypertension, while inhibition of the ACE/Ang II/AT1R signaling has been regarded as important pharmacological tools to prevent and treat vascular diseases such as hypertension [1, 3, 19]. Recently, most researches have been focused on the interactions between the ACE/Ang II/AT1R axis and miRNAs in hypertension. The miR-145, miR-27a/27b, and miR-483-3p have been shown to inhibit ACE expression by directly targeting the putative binding sites in the 3′-UTR of ACE transcripts [20–23]. There exists a negative feedback loop between miR-145 and ACE expression, mediating the switch of VSMC phenotypic differentiation to dedifferentiation. The ACE/Ang II signaling mediates various miRNAs expression via the activation of AT1R and these miRNAs, thereby contributing to VSMCs proliferation, cardiovascular remodeling, and hypertension (Figure 1) [24, 25]. In hypertensive status, Ang II-induced miR-130a mediated VSMCs proliferation and vascular remodeling by preventing the expression of growth arrest-specific homeobox (GAX) [11]. In human microvascular endothelial cells, exposure to Ang II increased the stability of HIF1-*α* with consequent induction of miR-138, which attenuated the bioavailability of NO and mediated the proinflammatory signal transduction [26]. Eskildsen et al. [27] reported that chronic Ang II infusion enhanced the levels of miR-132/-212 in hypertensive rats, which were prevented by treatment with AT1R blocker. Moreover, they observed that miR-132/-212 fine-tune multiple targets in Ang II signaling led to cardiac hypertrophy and fibrosis [28]. Ang II-mediated miRNAs have been identified to involve hypertension, including miR-19b, miR-29, miR-129-3p, miR-181b, and miR-483-3p (Table 1) [22–25, 29]. Kemp et al. [23] investigated that the miR-483-3p could target some components of RAAS directly. In their study, miR-483-3p regulated homeostatic levels of ACE, ACE2, and AT2R, indicating that this miRNA might function as an important regulator of manipulating RAAS. The intrinsic regulation of Ang II-responsive miRNAs on RAS components suggests that the modulation between Ang II and miRNAs is complicated (Table 1). In another work [30], miR-155 was found as a potent regulator of Ang II-induced VSMCs proliferation by inhibiting AT1R (Figure 1). In hypertensive patients, AT1R expression was negatively correlated with miR-155 expression and the interplay between miR-155 and AT1R was associated with the polymorphism (+1166A/C single-nucleotide polymorphism) located in the 3′-UTR of the AT1R [31]. Although the specific downstream targets of AT1R remain unclear, these findings suggest that miR-155 involve the Ang II/AT1R signaling and relate to vascular diseases.

## 4. The ACE2/Ang-(1-7)/Mas Signaling and MicroRNAs in Hypertension

The classical pathway of the RAS involving the ACE-Ang II-AT1 receptor axis is now antagonized by the second arm constituted by the ACE2-Ang-(1-7)-Mas receptor axis, exerting protective effects via modulating the inflammation, fibrosis, and vascular remodeling in hypertension [1–3]. Recent studies have shown the link between the ACE2/Ang-(1-7)/Mas signaling and miRNAs in hypertension (Table 1 and Figure 1) [32–34]. miRNA-targeting prediction algorithms revealed pupative binding sites for a variety of miRNAs in the 3′-UTR of ACE2 transcript, while only several miRNAs have been identified as regulators in inhibiting ACE2 expression [23, 32, 33]. In SHR model [33], exercise training significantly decreased the blood pressure levels and miR-143 expression levels in aortas in association with a drastic augment in circulating ACE2 and Ang-(1-7) levels, indicating the regulatory roles of miR-143 in the ACE2/Ang-(1-7) signaling (Table 1 and Figure 1). By establishing luciferase report system containing ACE2 3′-UTR, both miR-421 and miR-483-3p strikingly decreased ACE2 protein levels while loss of the two miRNAs reversed these effects, implying that miR-421 and miR-483-3p modulated ACE2 expression via translational repression rather than degraded the transcripts (Table 1) [23, 32]. In human aortic endothelial cells (Table 1), Ang-(1-7) decreased glycated albumin-mediated vascular inflammation and improved the vascular function through modulating the expression of miR-146a [34]. In addition, treatment with telmisartan modulated the level of miR-146a/b, along with the improvement of the ACE2/Ang-(1-7) levels and attenuation of vascular remodeling in hypertension [35–38]. Satoh et al. [39] reported that blockade of RAAS in coronary artery disease patients decreased protein levels of TLR4 and upregulated TLR4-responsive miRNAs expression including miR-31, miR-181a, miR-16, and miR-145 compared to controls. Although the related mechanism has not been identified, these researches permit speculation that RAAS blockades may exert cardiovascular protective effects by modulating the ACE2/Ang-(1-7) signaling and miRNAs levels. Taken together, there are potential interactions between the ACE2/Ang-(1-7) signaling and miRNAs in hypertension.

## 5. The Apelin/APJ Signaling and MicroRNAs in Hypertension

Apelin is a second catalytic substrate for ACE2 and functions as an inotropic and cardioprotective peptide [4, 38, 40–43]. The physiological effects of Apelin are exerted through binding to its receptor APJ, a seven-transmembrane G protein-coupled receptor that shares significant homology with the AT1R. Intriguingly, there is 54% sequence homology of the transmembrane regions between the APJ and AT1R and the anatomical distribution of both receptors and peptides overlaps in the cardiovascular system [42]. However, the effects of Apelin is independent of AT1 receptor and Ang II cannot bind to the APJ, although both of Ang II and Apelin can be degraded by ACE2 [38, 43]. The Apelin/APJ system has been shown to be involved in a wide range of pathophysiology effects in cardiovascular system, which is a necessary process in the initiation and development of various cardiovascular diseases such as pulmonary hypertension and essential hypertension [40, 41, 43]. Apelin levels were reduced in patients with essential hypertension independent of left ventricular systolic and diastolic dysfunction [44]. Treatment with Apelin has been shown to lower systolic blood pressure levels in hypertensive rats via a NO-dependent signaling [45, 46]. miRNAs are involved in VSMCs proliferation, cardiovascular remodeling, and the pathogenesis of hypertension (Table 1) [7, 16–18]. Ceylan-Isik et al. [47] found that treatment with Apelin strikingly prevented high fat diet-induced cardiac hypertrophy and contractile dysfunction associated with increased levels of miR-1, miR-208, and miR-133a. In addition, Apelin treatment significantly reduced the expression of fibroblast growth factor 2 (FGF2) and FGF receptor 1 (FGFR1) by regulating miR-424 and miR-503, contributing to the attenuation of VSMCs proliferation (Table 1 and Figure 1). Conversely, Apelin deficiency resulted in a marked increase in FGF2 expression and a reduction in expression of miR-424 and miR-503 that worsen pulmonary arterial hypertension in experimental models, suggesting the regulatory roles of Apelin in maintaining vascular homeostasis via the miR-424/503-FGF signaling [48]. Intriguingly, Apelin upregulated ACE2 in the failing hearts, coupled with upregulation of miR-424 and miR-503, whereas Ang-(1-7) administration improved cardiovascular dysfunction of Apelin-deficient mice in vivo [40, 48]. Moreover, the Apelin-APJ increased ACE2 activity and improved cardiovascular remodeling independently of AT1R signaling [40]. Both ACE2 and Apelin exhibit beneficial effects in the cardiovascular system, and recombinant ACE2 has recently been shown as a candidate therapeutic for treating hypertension in animal models [38, 40, 41]. The crosstalk between the ACE2/Apelin signaling and miRNAs provides an important mechanistic insight into hypertension and may lead to the development of new therapeutic regimens.

## 6. Conclusions, Limitations, and Perspectives

The discovery of the interaction between the ACE2/Apelin signaling and miRNAs during hypertension is an exciting affair in hypertensive research. ACE2 is a pleiotropic monocarboxypeptidase responsible for the conversion of Ang II to Ang-(1-7), thereby functioning as a negative regulator of the RAAS in cardiovascular system [3, 19, 38]. Apelin, as the second substrate of ACE2, exerts a cardiovascular protective effect including lowering blood pressure and improving cardiac contractility [4, 38–41]. The ACE2/Apelin signaling exhibits beneficial effects in the cardiovascular system and hypertension [3, 4, 38, 40]. The ACE2-coupled crosstalk among the RAAS, the Apelin system, and miRNAs provides an important mechanistic insight into hypertension.

Although most researches indicate the interactions among RAAS, ACE2/Apelin, and miRNAs in hypertension, these correlations are just in statistic level. These researches just found the linkage between the ACE2/Apelin and miRNAs in patients with hypertension or animal models of hypertension or* in vitro* VSMCs. However, the ACE2/Apelin-related miRNAs are really big families which contain “good” or “bad” components and may have additional control mechanisms beyond our current understanding. Thus, the corresponding targets and functional roles of the ACE2/Apelin-related miRNAs still need to be identified. The investigation of the relationship between the ACE2/Apelin signaling and miRNAs is just a beginning; there is still a long way to go. Further studies will be needed to explore the interactions and precise mechanisms of the ACE2/Apelin signaling and evaluate the role of validated and predicted targets of miRNAs in hypertension. Targeting the ACE2/Apelin signaling and miRNAs could lead to the development of a novel therapeutic approach for patients with hypertension and other vascular diseases related to cardiovascular remodeling.

## Figures and Tables

**Table 1:** The RAAS, Apelin/APJ signaling, and microRNAs in hypertension.

MicroRNAs	Targets	miRNA function	References
miR-143/145	Klf4; Klf5	Regulation of VSMCs proliferation	[7, 16, 17]
miR-365	Cyclin D1	Inhibition of VSMCs proliferation	[18]
miR-221/222	p27(Kip1); p57(Kip2)	Regulation of VSMCs differentiation and proliferation	[6, 7]
miR-126	VEGF; ICAM-1	Mediator of vascular dysfunction	[7, 18]
miR-221/222	Ets-1; VCAM-1; MCP-1	Regulation of inflammation and vascular remodeling	[7, 18]
miR-130a	GAX	Regulation of VSMCs proliferation	[11]
miR-145; -27a/27b;	ACE	Inhibition of ACE expression	[20–23]
miR-421; -143;	ACE2	Inhibition of ACE2 expression	[23, 32, 33]
miR-19b; -29; -132/212; -181b;	Ang II	Promotion of fibrosis and cardiovascular remodeling	[24–27, 29]
miR-146a	Ang-(1-7)	Inhibition of inflammation	[34]
miR-155	AT1R	Inhibition of VSMCs proliferation	[19, 30, 31]
miR-133a; -208; -1	Apelin/APJ	Regulation of cardiovascular hypertrophy and dysfunction	[47]
miR-424; -503	FGF2; FGFR1	Inhibition of VSMCs proliferation	[48]

RAAS: renin-angiotensin aldosterone system; Klf4: Krüppel-like factor4; Klf5: Krüppel-like factor5; p27(Kip1): cyclin-dependent kinase inhibitor 1B; p57(Kip2): cyclin-dependent kinase inhibitor 1B; VEGF: vascular endothelial growth factor; Ets-1: E26 transformation-specific sequence 1; VCAM-1: vascular cell adhesion molecule 1; MCP-1: monocyte chemoattractant protein 1; GAX: growth arrest-specific homeobox; ACE: angiotensin converting enzyme; ACE2: angiotensin converting enzyme 2; AT1R: Ang II type 1 receptor; FGF2: fibroblast growth factor 2; FGFR1: fibroblast growth factor receptor 1; VSMCs: vascular smooth muscle cells.
